# Evaluation of preventive, supportive and awareness building measures among international students in China in response to COVID-19: a structural equation modeling approach

**DOI:** 10.1186/s41256-021-00192-5

**Published:** 2021-03-13

**Authors:** Tanwne Sarker, Apurbo Sarkar, Md. Ghulam Rabbany, Milon Barmon, Rana Roy, Md. Ashfikur Rahman, Kh. Zulfikar Hossain, Fazlul Hoque, Muhammad Asaduzzaman

**Affiliations:** 1 School of Economics and Finance, Xi’an Jiaotong University, Xi’an, Shaanxi 710049 People’s Republic of China; 2 College of economics and management, Northwest A&F University, Yangling, Shaanxi 712100 People’s Republic of China; 3 Department of agribusiness and marketing, Sher-e-Bangla Agricultural University, Dhaka, 1207 Bangladesh; 4 Institute of Soil and Water Conservation, Northwest A&F University, Yangling, Shaanxi 712100 People’s Republic of China; 5 College of Natural Resource and Environment, Northwest A&F University, Yangling, Shaanxi 712100 People’s Republic of China; 6 Department of Agroforestry & Environmental Science, Sylhet Agricultural University, Sylhet, 3100 Bangladesh; 7 Centre for Global Health, University of Oslo, Kirkeveien 166, Frederik Holsts hus, 0450 Oslo, Norway

**Keywords:** COVID-19, Awareness building, Epidemic, PLS-SEM, Supportive measures, Trust, international student, modeling

## Abstract

**Background:**

Education institutions promptly implemented a set of steps to prevent the spread of COVID-19 among international Chinese students, such as restrictive physical exercise, mask wear, daily health reporting, etc. Success of such behavioral change campaigns largely depends on awareness building, satisfaction and trust on the authorities. The purpose of this current study is to assess the preventive, supportive and awareness-building steps taken during the COVID-19 pandemic for international students in China, that will be useful for planning such a behavioral change campaign in the potential pandemic situation in other parts of the world.

**Methods:**

We conducted an online-based e-questionnaire survey among 467 international students in China through WeChat. The data collection duration was from February 20, 2020 to March 10, 2020 and we focused on their level of awareness, satisfaction, and trust in authorities regarding pandemic measures. Simple bivariate statistics was used to describe the background characteristics of the respondents along with adoption of the partial least squares-structural equation modeling **(**PLS-SEM) as the final model to demonstrate the relationship between the variables.

**Results:**

In our study, the leading group of the respondents were within 31 to 35 years’ age group (39.82%), male (61.88%), living single (58.24%) and doctoral level students (39.8%). The preventive and supportive measures taken by students and/or provided by the respective institution or authorities were positively related to students’ satisfaction and had an acceptable strength (β = 0.611, t = 9.679, *p* < 0.001). The trust gained in authorities also showed an acceptable strength (β = 0.381, t = 5.653, *p* < 0.001) with a positive direction. Again, the personnel awareness building related to both students’ satisfaction (β = 0.295, t = 2.719, *p* < 0.001) and trust gain (β = 0.131, t = 1.986, *p* < 0.05) in authorities had a positive and acceptable intensity. Therefore, our study clearly demonstrates the great impact of preventive and supportive measures in the development of students’ satisfaction (R^2^ = 0.507 indicating moderate relationship). The satisfied students possessed a strong influence which eventually helped in building sufficient trust on their institutions (R^2^ = 0.797 indicating above substantial relationship).

**Conclusions:**

The worldwide student group is one of the most affected and vulnerable communities in this situation. So, there is a profound ground of research on how different states or authorities handle such situation. In this study, we have depicted the types and magnitude of care taken by Chinese government and educational institutions towards international students to relieve the panic of pandemic situation. Further research and such initiatives should be taken in to consideration for future emerging conditions.

## Introduction

The Novel Coronavirus Disease 2019 (COVID-19) is the deadliest pandemic of this century having the most disseminated outbreak over a wide geographical area. Several reports have indicated that there could be multi-starting points for the outbreak of COVID-19, while Wuhan is the first city in China to detect and suffer from this fatal virus [1–3]. The increasing number of infected persons caused a severe threat to public health status, including international students living in China. The World Health Organization (WHO) declared the 2019-nCoV outbreak as the sixth emergency public health of global concern [4]. The COVID-19 is considered so destructive and contagious due to its rapid spread from a small city to global community [5, 6].

In order to reduce the rapid spread and adverse health impacts, increasing public awareness in such conditions is of great importance. On January 26, 2020, China initiated level-1 public health response for its 30 provinces [7] which means the provincial headquarters will organize and coordinate the emergency response. They will be working within their administrative territory based on a unified decision disseminated by the State Council during any severe public health emergency. In addition, the Chinese government has been utilizing several communication methods to disseminate and update timely reports and provide preventive advice to the public in such circumstances.

Largely, the success of such initiatives depends on the change of health seeking behaviour and attitudes of the public. The theory of planned behavior, people’s perceptions and behavioral intentions are major critical factors affecting and understanding of their actual behavior [8]. The outcome of these initiatives is somewhat challenging to measure for the non-native people due to many factors like social, cultural, linguistic or building trust. Thus, the need of people’s perceptions, especially the perceptions of international students living in China about COVID-19, is very crucial during the current epidemic situation. That is why; we focused our concentration on significant gaps in knowledge and existing perceptions among them towards this COVID-19 outbreak.

During epidemic conditions, taking preventive measures (such as reducing outdoor activities and wearing N95 masks) can diminish the threat to public health [9]. Along with these, supportive measures taken by the institution can significantly decrease disease contamination. Hence, it is essential to examine the factors associated with the intentions of international students to take up these preventive measures as well as supports provided by their respective institutions to provide safety and satisfaction during epidemic conditions. The following hypotheses are suggested based on the above-mentioned discussion:

- H1: Preventive and supportive measures taken by students and/or provided by the respective institution or authorities are positively related to students’ satisfaction.
- H2: Preventive and supportive measures taken by students and/or provided by the respective institution or authorities are positively related to gain trust in authorities.

Personnel awareness levels in terms of knowledge concerning health hazards play a significant function in the management of risk communication research. Knowledge theory is a widely used framework for building awareness which indicates that individuals’ response in terms of risk is conditioned by their knowledge level [10]. The better risk knowledge a person possesses, the more appropriate risk judgments can be gained during epidemic situations. A wealth of literature recommends that mass media plays a significant role in disseminating information to enrich public awareness of health and contingent circumstances [11–15]. The more people depend on mass media to get information, the more attention they will pay to the news generated by these media outlets, and thus the more likely their behaviors and attitudes will be changed or strengthened [16, 17]. Moreover, it was also found that the increasing awareness level causes a notable decline in Ebola virus disease transmission [18]. Based on the above discussion, we suggest the following two hypotheses:

- H3: Personnel awareness building is positively related to students’ satisfaction.
- H4: Personnel awareness building is positively related to gain trust in authorities.

Communication strategy is crucial for controlling the epidemic, affects the consequence of epidemic management, control, and public trust. Epidemic associated information must be conveyed to the citizens in such a way that construct, maintain and restore trust and respect to local cultures and country norms [19, 20]. Therefore, we propose the following hypothesis:

- H5: Students’ satisfaction is positively related to gain trust over authorities.

The international student community in China, one of the largest sets in the world, is concerned about the COVID-19 pandemic. During any kinds of pandemic population group like international students are generally afraid of the lack of proper instructions and supports from the relevant duty bearer, which may be family or government or educational institutions etc. Because, they are living in a place where the outbreak has emerged, and far away from their families and country. In addition, they were indulged with a very vulnerable situation as government usually cannot impose such strict policy as they do with their own inhabitants. Again, information regarding such incidents sometimes may pass through some extensive filtration, which can mostly affect the satisfaction and trust as well. So, there is a stronger ground of research on how the Chinese government handles this dilemmatic situation with the help of preventive, supportive, and awareness building mechanisms. In addition, very limited information is in place that can profoundly trigger linkages between these variables and make some substantial recommendations for other countries to make use of awareness-building improvements through preventive and supportive measures. As the international student community is not well aware of strict internal policies and is mostly managed by their institutions, our current research aims to bridge the gap in research on how the preventive, supportive and awareness-building mechanism triggers the level of satisfaction of international students in the face of this Covid-19 pandemic. In the course of our study, we refer a set of regulatory materials such as prevention leaflets, usage guidelines of preventive tools and materials, daily supply list of food, and other hygienic materials with the full supports from the College of International Educations of investigated Universities. Moreover, the researchers also rendered their efforts to form and actively observe several International groups via various social media platforms like WeChat, web no, QQ, and especially WhatsApp and Facebook to gather authentic sources of information regarding satisfaction level. The evaluation could be essential for other countries and future but ethical research as it comprises some mostly used factors that render preventive, supportive, and awareness building mechanisms.

## Methods

### Setting and sample

Empirical data has been extracted from an online survey from February 20 to March 10, 2020, among international students studying in different universities in China. Mostly international students who are enrolled and staying in different universities across China were the target population. An online-based e-questionnaire (Table 1) was used to collect information associated with the research questions and objectives. A Seven-point Likert-type scale was used in this study, where 1 is set for strongly disagree and 7 for strongly agree. Prior to distributing the questionnaire, the Snowball sampling technique was utilized in order to find out the educational institutions where international students are staying and their WeChat groups. An informed consent form was attached to the e-questionnaire, and each participant was asked through this form to submit his or her consent before filling up the e-questionnaire. The most familiar social media platform in China, WeChat, was used to distribute the students’ e-questionnaire.

**Table 1:** The excerpts of the questionnaire utilized in this research

Variables	Excerpts questions of Indicators	Model
Preventive and supportive measures	I am satisfied with the proper use of mask and hand gloves that can prevent this infection	P&S_1
	I am satisfied with avoids public transport and gathering in the last 1 month that can prevent this infection	P&S_2
	I am satisfied with the use of hand sanitizer, alcohol, and chemicals that can prevent this infection	P&S_3
	I am satisfied with current food preparation and consumption that can prevent this infection	P&S_4
	I am satisfied with my regular exercise which is instructed by authorities to protect me from this infection	P&S_5
	I am satisfied with the preventive measure taken by me to avoid direct contact with the animal to protect from this infection	P&S_6
	I am satisfied to participate in online class supported by an institution that can prevent this infection	P&S_7
	I am satisfied with authorities support to restrict movement that can protect me from this infection	P&S_8
	I am satisfied with the establishment of the temporary market and regular supplies of food and medicine by the authorities	P&S_9
	I am satisfied with maintaining register book for body temperature during exit and entre point by the authorities	P&S_10
	I am satisfied with regular health update collected by the institution	P&S_11
	I am satisfied with extra care taken the authorities for the international student during this period	P&S_12
Awareness building	I am aware of regular hand washing during this period	AW_1
	I am aware of my health to protect from cold during this period	AW_2
	I am careful about not to frequent face touch	AW_3
	I am aware of building immunity system through physical exercise	AW_4
	I am aware of participating in awareness activities arranged by the institution to protect from this infection	AW_5
	I am aware of the rumor and symptom of this epidemic	AW_6
	I am aware of involvement in mass media regarding this epidemic	AW_7
Trust in authorities	Overall, I trust my institutions that they will protect me from this infection	TR_1
	Overall, I trust the local authorities that they will protect me from this infection	TR_2
	Overall, I trust on Chinese Government that they will protect me from this infection	TR_3

**Fig. 1:**
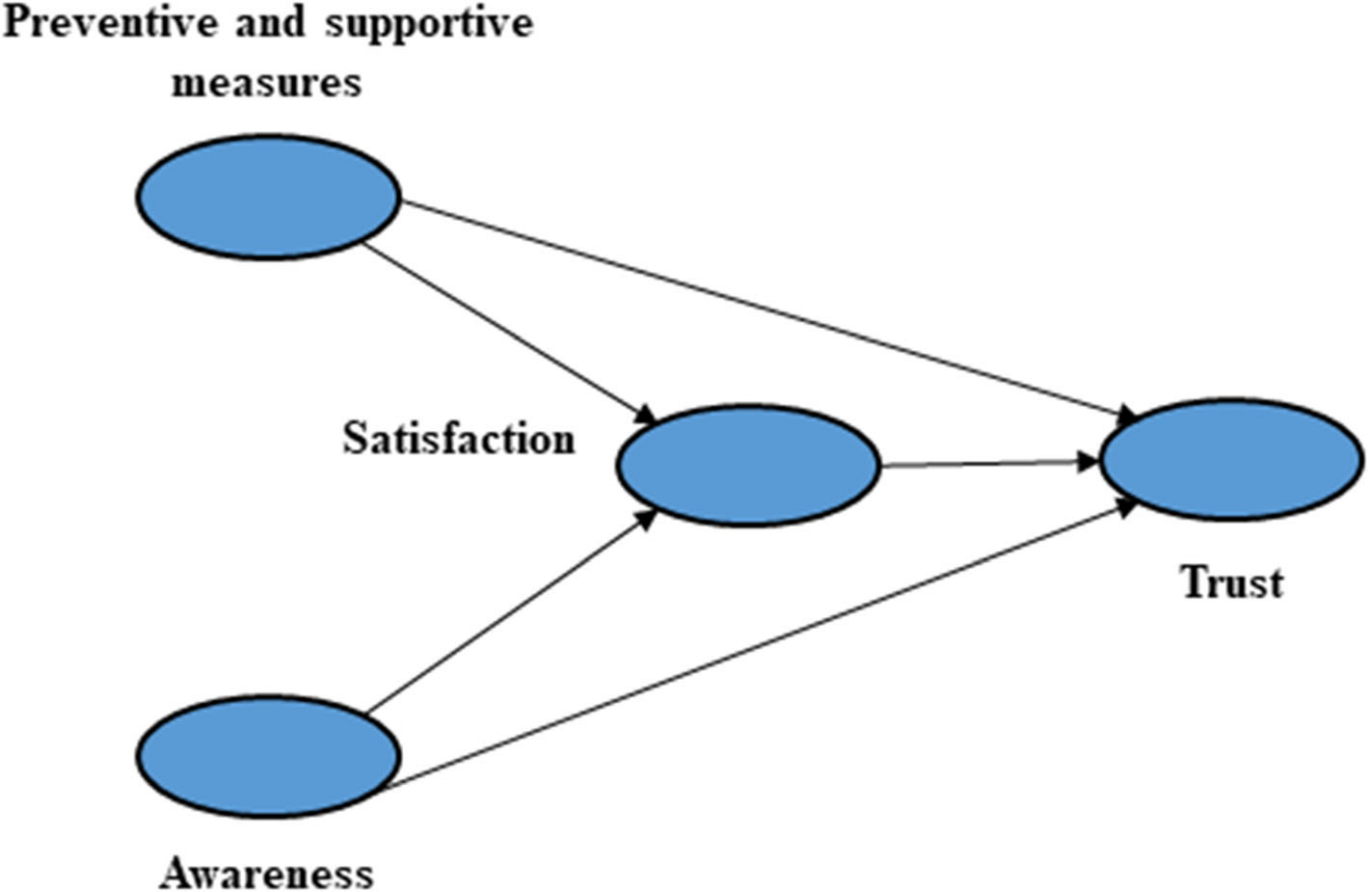
The Conceptual model for PLS-SEM

### Research model

The theoretical outline, which is portrayed according to the SEM tactic, is shown in Fig. 1. The theoretical outline is mainly focused on the establishment and combinations of preventive and supportive measures, awareness building, and trust in authorities.

**Fig. 2:**
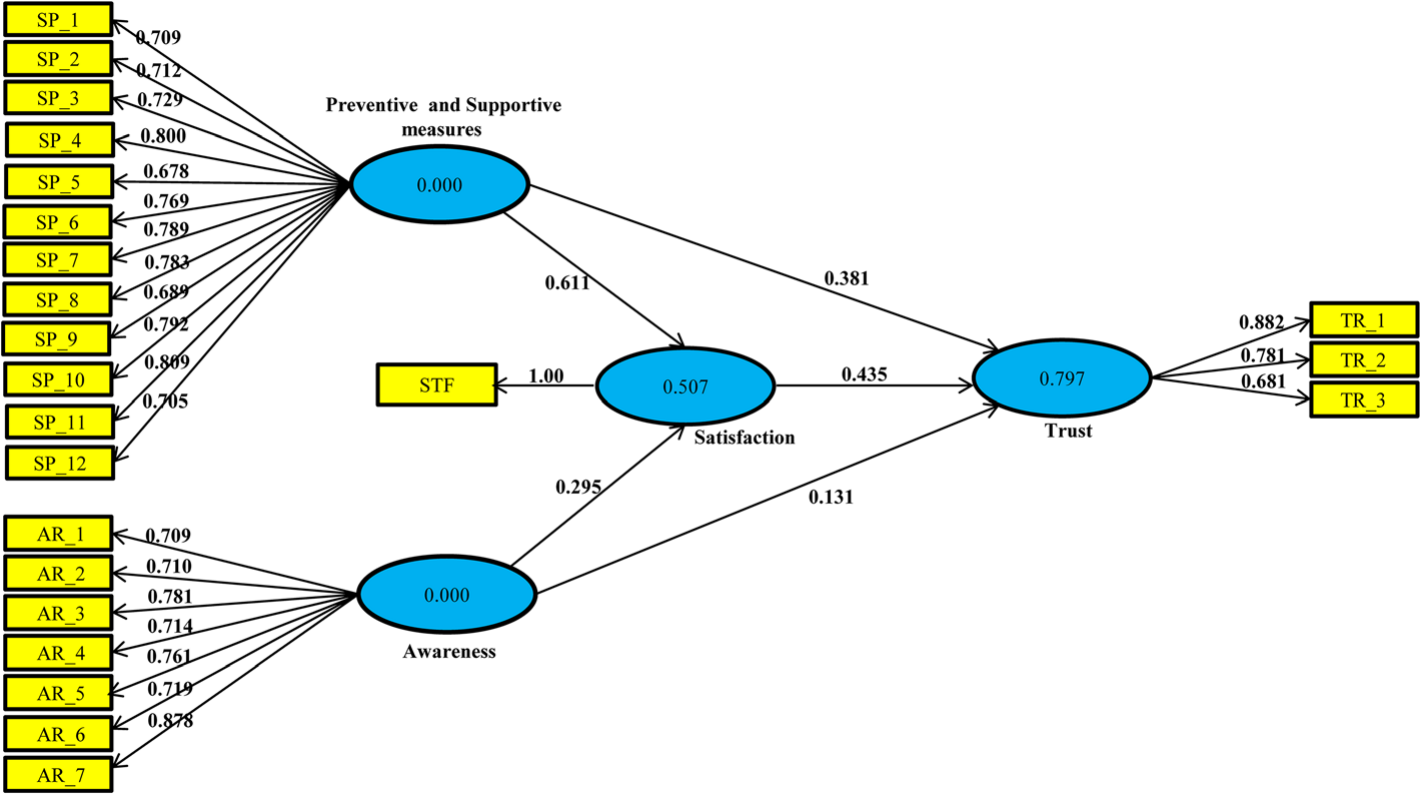
A structural model for PLS-SEM hypothesis tests

### Data collection and processing

All the co-authors related to this study were contacted through WeChat with the international students in different educational institutions in order to distribute the e-questionnaire. We found 52 WeChat groups from 52 universities in China, circulated the e-questionnaire, and requested them to share the e-questionnaire with their friends studying in their respective universities. Students from 52 different universities participated in this online survey. Total 467 international students fulfilled the e-questionnaire and submitted it. After reading the consent, 16 students refused to participate the e-questionnaire. The final data has been processed through the Smart PLS software (version 2.0).

### Analysis

Structural equation modeling is designed with the two-phase model (i) measurement model and (ii) structural model. The inner relationships between latent variables and observed variables are portraits in the measurement model, and the latter with the structural model is used to investigate the loadings and estimating indicators [21, 22].

## Results

### Demographic analysis

Total 467 students have participated in this study (Table 2). Among them, 289 (61.88%) participants were males. The age group of 31 to 35 years was leading, with 186 (39.82%) entries, followed by the age group of 26 to 30 years, with 140 (29.97%) entries. The majority of the students were living single 272 (58.24%), followed by with partner 117 (25.05%), and family with children 78 (16.7%). In terms of educational level, participants were categorized into four groups: (i) Post-Doctoral, 47 (10.06%); (ii) Doctoral, 186 (39.8%); (iii) Masters, 140 (29.97%) and (iv) Bachelor, 94 (20.12%). Students from 21 countries participated in the study including more than half of them were from Pakistan, 247 (52.89%).

**Table 2:** Demographic attributes of the respondents

Attributes	Distribution	Frequency	Percent (%)
Gender	Male	289	61.88
	Female	178	38.11
Age	Under 25 years	94	20.12
	26–30 years	140	29.97
	31–35 years	186	39.82
	36–40 years	34	7.28
	Above 40 years	13	2.78
Family pattern	Single student	272	58.24
	With family (husband/wife)	117	25.05
	Family with children	78	16.70
Educational Level	Post-Doctoral	47	10.06
	Doctoral	186	39.8
	Master’s	140	29.97
	Honor’s	94	20.12
Country of origin	Pakistan	247	52.89
	Kazakhstan	28	5.99
	Mongolia	26	5.56
	Bangladesh	22	4.71
	Egypt	18	3.85
	Vietnam	16	3.42
	Cambodia	15	3.21
	Thailand	12	2.56
	India	12	2.56
	Srilanka	12	2.56
	Tanzania	9	1.92
	Sudan	8	1.71
	Laos	8	1.71
	Nigeria	7	1.49
	Denmark	6	1.28
	Myanmar	5	1.07
	Ethiopia	4	0.86
	Uganda	3	0.64
	Poland	2	0.42
	Turkistan	4	0.86
	Uzbekistan	3	0.64

### PLS-SEM algorithm

The 22 indicators of the conceptual framework model were run with the help of Smart PLS 2.0 version software and the structural framework used in the hypothesis testing parts is illustrated in Fig. 2. Note that the preventive and supportive measures’ paradigm had twelve indicators, the awareness-building paradigm had seven indicators, and the trust paradigm had three indicators. The initial assessments encompass the metrics with measurement characteristics of the outer framework, which represents the paradigms and their construction described in the PLS-SEM framework [23]. Smart PLS comprises a set of standard metrics like indicator loadings, composite reliability, average variance extracted (AVE), path coefficients, inner construct correlations, latent variable scores, *t*-values, and so on. A structural procedure of investigating the loadings and eliminating indicators (with loadings < 0.70) was adopted [24]. The leading step during the evaluation of a PLS-SEM framework was to investigate the outer model to facilitate the exertion and validation of the model dimension. For this reason, inner-relationships among the paradigms and their indicators were measured. Table 3 shows the composite reliability wide-ranging between 0.87 to 0.90 for the four paradigms, which are far greater than the minimum requirement of 0.7, as proposed by Hair et al. [24].

**Table 3:** PLS-SEM average variance extracted composite reliability and R^2^ for endogenous constructs

Construct	Indicators	Loadings	Composite Reliability	Average variance extracted (AVE)	R^2^
Preventive and supportive measures	S&P_1	0.709	0.8981	0.731	
	S&P_2	0.712			
	S&P_3	0.729			
	S&P_4	0.800			
	S&P_5	0.678			
	S&P_6	0.769			
	S&P_7	0.789			
	S&P_8	0.783			
	S&P_9	0.689			
	S&P_10	0.792			
	S&P_11	0.809			
	S&P_12	0.705			
Students satisfaction			0.8956	–	0.507
Awareness-building	AW_1	0.709	0.8739	0.626	
	AW_2	0.710			
	AW_3	0.781			
	AW_4	0.714			
	AW_5	0.761			
	AW_6	0.719			
	AW_7	0.878			
Trust in authorities	TR_1	0.882	0.8923	0.781	0.797
	TR_2	0.781			
	TR_3	0.681			

The lowest average variance extracted (AVE) for all the constructs of our paper exceeded the minimum accepted value of 0.5 [25], representing the adequate convergent validity Furthermore, for convergent validity, the composite reliability is higher than the AVE values of each and every variable which represents the convergent validity of the current model. Table 4 shows the AVEs of the diagonal and the squared inner construct correlations off the diagonal. The Fornell–Larcker criterion [26] displayed that all AVEs were greater than the squared relationships of the inner construct.

**Table 4:** PLS-SEM Fornell –Larcker test for discriminant validity

	Protective and supportive measures	Awareness-building	Students’ satisfaction	Trust in authorities
Protective and supportive measures	**0.731**			
Students’ satisfaction	0.360	**0.626**		
Awareness-building	0.581	0.410	**Single item construct**	
Trust in authorities	0.474	0.563	0.529	**0.781**

The hypotheses aimed for the current research were also verified by the bootstrapping resampling procedure with 200 repetitions. Bootstrapping is a nonparametric method that makes no distributional notion of the variables, facilitates with an estimated value of standard errors and the assurance intermissions, and tests the study hypotheses. The hypothesis H1 (preventive and supportive measures taken by students and/or provided by the respective institution or authorities are positively related to students’ satisfaction) had an acceptable strength (β = 0.611, t = 9.679, *p* < 0.001) and a positive direction (presented in Table 5). The hypothesis H2 (Preventive and supportive measures taken by students and/or provided by the respective institute or authorities are positively related to gain trust in authorities) showed an acceptable strength (β = 0.381, t = 5.653, *p* < 0.001) and a positive direction. The hypothesis H3 (personnel awareness building is positively related to students’ satisfaction) had an acceptable intensity (β = 0.295, t = 2.719, *p* < 0.001) and a positive direction. The hypothesis H4 (personnel awareness building is positively related to gain trust in authorities) generated an acceptable concentration (β = 0.131, t = 1.986, *p* < 0.05) and a positive direction. Finally, the fifth hypothesis (students’ satisfaction is positively related to gain trust in authorities) had an acceptable intensity (β = 0.435, t = 7.135, *p* < 0.001) and a positive direction.

**Table 5:** Result of hypothesis tests based on PLS-SEM based model

Hypothesis	Hypothesis Path	Path Coefficient	T-Values	Accept or reject the significance
H1	S&P → Satisfaction	0.611	9.679	Accept***
H2	S&P → Trust	0.381	5.653	Accept***
H3	Awareness → Satisfaction	0.295	2.719	Accept***
H4	Awareness → Trust	0.131	1.986	Accept**
H5	Satisfaction → Trust	0.435	7.135	Accept***

Critical *t*-values for a two-tailed test are: < 1.96 (*p* > 0.05*), 1.96 (*p* = 0.05**), and 2.58 (*p* = 0.001***)

We also examined the R^2^ values for the two endogenous paradigms, students’ satisfaction and trust in authorities. R^2^ can be categorized into one of three classifications for social science studies: weak (0.25), moderate (0.50), or substantial (0.75) [24]. Prediction of students’ satisfaction, the key outcome degree of the model, was nearly moderate, with an R^2^ = 0.507. Prediction of trust in authorities was above substantial, with an R^2^ = 0.797. The extents of the R^2^ values for endogenous and exogenous paradigms were measured significant for construal determinations within the study.

## Discussion

The COVID-19 came into sight in Wuhan just 1 month prior to the spring festival of China and a huge population movement during this period caused significant challenges for prevention and controlled the spread of infections. Therefore, it spread out rapidly from Hubei to whole China**.** The COVID-19 can transmit from human to human, and no effective drug has been invented yet. The most efficient preventive and control ways are to identify suspected and confirmed patients and keep them isolated while, personal protection as means of hygienic practice must be taken. Hence, increasing protective measures and awareness building are an important measure adapted and suggested by health practitioners and authorities to reduce and prevent the high transmission rate of this deadly virus.

In our study, the analytical method produced robust results and confirmed that the students’ satisfaction found as a meaningful partial mediator. The results from the PLS-SEM analyses showed that- a large amount of the variance in the endogenous construct trust (80%) is explained by the three constructs of preventive and supportive measures taken by students and respective authorities, personal awareness-building and students’ satisfaction. Trust over government has long been considered as a vital factor of citizens’ compliance with public health policies, particularly during epidemic conditions which is endorsed by the previous study of Blair et al. [27] and documented that that supportive measures and policies taken by the Liberia government to control the Ebola virus disease epidemic were positively associated with gaining public trust over authorities.

In terms of the strength of the relationships, the PLS-SEM model revealed a strong and significant relationship between preventive and supportive measures taken by students and/or provided by the respective institutions or authorities lead to trust over authorities (0.381) (Fig. 2; Table 5). The possible explanation could be during this pandemic Chinese central and local governments has taken several effective measures promptly. Such as, Chinese health authorities did an urgent investigation in the most affected areas to rapidly characterize the disease and patient intending to keep confirmed and suspected patients in strict isolation, examining of clinical contact status of the patients, and developing rapid diagnostic and treatment processes [28].

In line with this, on January 23, 2020, the local authorities of Wuhan declared the suspension of all kinds of public transportation, including highways, bus stations, railway stations and airports in the city, preventing further disease transmission. Consequently, most of the provinces in China declared a “Level I Emergency Response” by adopting a series of measures followed by Wuhan strategies. Furthermore, several compulsory measures like restrict mobility, prohibited mass gatherings, shutdown school, were taken place alongside online schooling and working-from-home were encouraged and forced with a view to decreasing the public transmission [29].

The PLS-SEM model revealed a strong and significant relationship between personal awareness and means of gaining trust over authorities (0.131) (Fig. 2; Table 5). In this regard Chinese government tried to increase public awareness through publicizing regular updates about surveillance and confirmed cases on different websites and social media [30]. in line with this psychologists and psychiatrists using the internet and social media (e.g., WeChat, Weibo, etc.) to make aware of the public dealing with psychological stress. An expert from Peking University Sixth Hospital of China made several suggestions for the general people to manage mental stress. These involved judging the accuracy of information disclosed, developing social support systems (e.g., friends and families), eradicating stigma linked with the epidemic, maintaining a healthy life under safe conditions, and using the psychosocial service system, mainly telephone- and internet-based counselling for health-care staff, infected patients, family members and the public [31].

Satisfaction depends on whether one has sympathy for what the authorities do and whether one thinks, what the authorities are doing is good for society. Previous studies documented that a positive relationship remains between satisfaction and trust over the government [32, 33].

The present study found a strong relationship between students’ satisfaction and trust in authorities (Fig. 2; Table 5). The possible reason behind this may be the international students living in China during COVID-19 found their respective institutions and relevant authorities did their best to control this pandemic and trying to keep them safe from being infected.

Despite our enormous efforts, our study has some limitations. It is possible that communal desirability apprehensions can lead the responses to our questionnaire with some extend of misperception. We reduced these concerns by avoiding the use of a brief discussion and pilot test. Moreover, our findings are not considered identical because the respondents of our study are only foreign students. Most notably, we found some extensive-expression of conspiracy belief in our prospective set of respondents who has some extend of obligatory for the institutions and authorities. So, there might be some biased responses. We tried to minimize this by a close discussion with some respondents and compiled those in our analysis. The linkage between satisfaction and trust in terms of such epidemics has limited empirical pieces of evidence, and the interconnection is relatively complex. Lastly, future researchers should investigate whether these findings vary in various situations and country settings. In this study, we do not test these variants in fear of losing focus on our core objectives and it could lead theoretically assorted treatment based on sources satisfaction and trust, which needs further statistical analysis. Notwithstanding these limits, this is the first study on COVID-19, which used SEM to assess behaviour change.

Not surprisingly, the findings of our study triggered a positive relationship between preventive and supportive measures towards shaping the satisfaction level and eventually building trust in institutions. The results are entirely parallel with the findings of Aristovnik et al. and Paek et al. [34, 35] that the awareness building is a predictor to gain the satisfaction of any individuals, any institutions can gain trust and quantify students’ satisfaction by practicing the awareness building, as stated by Valenzuela et al. [36], which is also proved by our studies hypothesis test. According to Prati et al. [37], it is quite evident if any person has a certain amount of satisfaction over any course of action of institutions, he or she might have been possessed a particular course of trust over the institutions, which is one of the prime findings of our study too.

## Conclusion

Overall, the findings revealed that a strong and significant relationship between preventive and supportive measures taken by students and/or provided by the respective institutions or authorities lead to trust over authorities. Chinese government as well as educational institutions regularly updated and monitor the pandemic situation and responded appropriately like restriction of mobility, prohibition of mass gatherings, shutdown school, encouraging work from home, which virtually increased awareness, satisfied students and helped to gain trust on authorities. Therefore, to advise preventive and supportive measures, awareness building through providing trustworthy information should be given priority.

## Data Availability

The dataset used and analyzed during the current study is available from the Corresponding authors on reasonable request.

## Abbreviations

PLS-SEM: Partial least squares-structural equation modeling
COVID-19: Coronavirus disease 2019
AVE: Average variance extracted
SEM: Structural equation modeling
WHO: World Health Organization
